# Activity-Based Anorexia Reduces Body Weight without Inducing a Separate Food Intake Microstructure or Activity Phenotype in Female Rats—Mediation via an Activation of Distinct Brain Nuclei

**DOI:** 10.3389/fnins.2016.00475

**Published:** 2016-10-25

**Authors:** Sophie Scharner, Philip Prinz, Miriam Goebel-Stengel, Peter Kobelt, Tobias Hofmann, Matthias Rose, Andreas Stengel

**Affiliations:** ^1^Division of Psychosomatic Medicine, Charité Center for Internal Medicine and Dermatology, Charité-Universitätsmedizin BerlinBerlin, Germany; ^2^Department of Internal Medicine and Institute of Neurogastroenterology, Martin-Luther-Krankenhaus BerlinBerlin, Germany

**Keywords:** anorexia nervosa, body weight, brain-gut axis, eating disorder, Fos, psychosomatic, wheel running

## Abstract

Anorexia nervosa (AN) is accompanied by severe somatic and psychosocial complications. However, the underlying pathogenesis is poorly understood, treatment is challenging and often hampered by high relapse. Therefore, more basic research is needed to better understand the disease. Since hyperactivity often plays a role in AN, we characterized an animal model to mimic AN using restricted feeding and hyperactivity. Female Sprague-Dawley rats were divided into four groups: no activity/*ad libitum* feeding (*ad libitum*, AL, *n* = 9), activity/*ad libitum* feeding (activity, AC, *n* = 9), no activity/restricted feeding (RF, *n* = 12) and activity/restricted feeding (activity-based anorexia, ABA, *n* = 11). During the first week all rats were fed *ad libitum*, ABA and AC had access to a running wheel for 24 h/day. From week two ABA and RF only had access to food from 9:00 to 10:30 a.m. Body weight was assessed daily, activity and food intake monitored electronically, brain activation assessed using Fos immunohistochemistry at the end of the experiment. While during the first week no body weight differences were observed (*p* > 0.05), after food restriction RF rats showed a body weight decrease: −13% vs. day eight (*p* < 0.001) and vs. AC (−22%, *p* < 0.001) and AL (−26%, *p* < 0.001) that gained body weight (+10% and +13%, respectively; *p* < 0.001). ABA showed an additional body weight loss (−9%) compared to RF (*p* < 0.001) reaching a body weight loss of −22% during the 2-week restricted feeding period (*p* < 0.001). Food intake was greatly reduced in RF (−38%) and ABA (−41%) compared to AL (*p* < 0.001). Interestingly, no difference in 1.5-h food intake microstructure was observed between RF and ABA (*p* > 0.05). Similarly, the daily physical activity was not different between AC and ABA (*p* > 0.05). The investigation of Fos expression in the brain showed neuronal activation in several brain nuclei such as the supraoptic nucleus, arcuate nucleus, locus coeruleus and nucleus of the solitary tract of ABA compared to AL rats. In conclusion, ABA combining physical activity and restricted feeding likely represents a suited animal model for AN to study pathophysiological alterations and pharmacological treatment options. Nonetheless, cautious interpretation of the data is necessary since rats do not voluntarily reduce their body weight as observed in human AN.

## Introduction

Anorexia nervosa (AN) is an eating disorder characterized by the desire to lose body weight or to maintain body weight at a lower level than normal for age and height. Moreover, patients suffer from an intense fear of gaining weight and a body image disturbance (American Psychiatric Association, 2013). AN has a high prevalence in adolescent girls and young women (Nagl et al., 2016); the lifetime prevalence for AN in European women was reported to be 0.9% (Preti et al., 2009), similar levels were reported for the United States (Hudson et al., 2007). The treatment of AN is challenging and mostly comprised of structured care and psychotherapy (Zipfel et al., 2014); however, treatment is hampered by a high relapse rate (Herzog et al., 1997; Zipfel et al., 2015). While only about half of the patients recover, one third improves but continues to have symptoms and 20% remain severely chronically ill (Steinhausen, 2002). Lastly, AN has a considerable weighted mortality rate (deaths per 1000 person-years) of 5.1 (Arcelus et al., 2011). It is to note that although AN is clinically well characterized, the pathogenesis underlying the disease is still not well established. Moreover, no specific pharmacological treatment is available. Therefore, more research is needed to better characterize the disease and to identify possible new treatment targets.

Progress in medical research is often achieved by establishing an animal model of a disease that can help to investigate the underlying pathophysiology. It was already in 1967 when Routtenberg and Kuznesof observed that rodents tend to self-starvation when exposed to a time-restricted feeding schedule and given the possibility of voluntary physical activity in a running wheel (Routtenberg and Kuznesof, 1967). As hyperactivity can be observed in a considerable subset (ranging from 31 to 80%) of patients with AN (Davis et al., 1997), animal models using physical activity mimic this condition. The combination of a restricted feeding schedule and the access to physical exercise using a running wheel has been used to mimic features of human AN; the model was termed activity-based anorexia (Casper et al., 2008).

Subsequently, the model has been largely characterized and several alterations observed such as an increased brain γ-aminobutyric acid (GABA) (Aoki et al., 2012) and endocannabinoid signaling (Casteels et al., 2014), disturbances in food-anticipatory dopamine and serotonin release (Verhagen et al., 2009) along with an involvement of several food intake-regulatory hormones, e.g., ghrelin (Legrand et al., 2016) and leptin (Hillebrand et al., 2005b) and lastly, an activation of the hypothalamus-pituitary-adrenal axis (Burden et al., 1993), changes that might play a role in human AN as well. These alterations are likely to be involved in several changes observed: besides a reduction in food intake and body weight also an intestinal barrier dysfunction (Jésus et al., 2014), a disruption of neural development in the hippocampus (Chowdhury et al., 2014) and an impairment of memory function (Paulukat et al., 2016), increased anxiety (Kinzig and Hargrave, 2010) and the development of stress ulcers (Doerries et al., 1991), features also observed (Kline, 1979; Ghadirian et al., 1993; Swinbourne and Touyz, 2007; Huber et al., 2015; Kjaersdam Telleus et al., 2015) or suspected in patients with AN. Taken together, activity-based anorexia—despite the major limitation of being an animal model merely mimicking features of a disease—is likely a suited tool to study aspects of the pathogenesis of human AN.

The aim of the present study was first to establish the model of activity-based anorexia in our laboratory investigating food intake, running wheel activity and body weight in female rats. Only female rats were used due to the higher prevalence of anorexia in females compared to males (Steinhausen and Jensen, 2015). Next, we investigated the food intake microstructure underlying the reduction in food intake in this animal model using an automated food intake monitoring system recently established for the use in rats (Teuffel et al., 2015). To further characterize possible underlying alterations in brain activity we used the neuronal expression marker Fos and performed a brain mapping in rats subjected to activity-based anorexia.

## Materials and methods

### Animals

Female Sprague-Dawley rats (Harlan-Winkelmann Co., Borchen, Germany) weighing 150–180 g upon their arrival were housed in groups under conditions of controlled illumination (12:12 h light/dark cycle, lights on/off: 06:00 a.m./06:00 p.m.) and temperature (21–23°C). Rats were fed with standard rat chow (ssniff Spezialdiäten GmbH, Soest, Germany) and tap water *ad libitum* unless otherwise specified. This study was carried out in accordance with the recommendation of the institutional guidelines; the protocol was approved by the state authority for animal research (#G 0117/14).

### Activity-based anorexia

After an initial acclimatization period of 7 days, rats (total *n* = 44) were randomly assigned to one of four groups: (a) *ad libitum* group: no extra activity + *ad libitum* feeding schedule, (b) activity group: voluntary activity in a running wheel + *ad libitum* feeding schedule, (c) restricted feeding group: no extra activity + restricted feeding schedule, and (d) activity-based anorexia group: voluntary activity in a running wheel + restricted feeding schedule.

During the first week of the experiment, all rats were fed *ad libitum* and separated into single housing cages which were placed adjacent to each other to provide sight, acoustic and odor contact. Rats of the activity and activity-based anorexia group had access to a running wheel inside the cage for 24 h/day, while the sedentary groups (*ad libitum* and restricted feeding group) were housed without running wheel under otherwise identical conditions. All cages contained environmental enrichment and bedding material. Rats were acclimated to their new cages for 1 week and handled daily to become accustomed to the interaction with the investigator. This included daily removal of the rat from the cage to measure body weight. The daily routine was performed between 08:00 and 09:00 a.m.

Food restriction conditions started on day eight of the experiment. Rats of the restricted feeding as well as activity-based anorexia group received food from 09:00 to 10:30 a.m. (the 90-min feeding period during the light phase was based on Luyten et al., 2009; Wu et al., 2014), while the other two groups (*ad libitum* and activity group) continued to have access to food for 24 h/day. Body weight, food intake and activity were monitored over a period of 21 days. The experiment was discontinued and animals euthanized when the body weight loss exceeded 25%.

## Measurements

### Monitoring of body weight

Rats were weighed daily between 08:00 and 09:00 a.m. Body weight and body weight changes were calculated for the whole 21-day experimental period (1 week of *ad libitum* food intake and 2 weeks of restricted feeding).

### Monitoring of food intake and food intake microstructure

The microstructural analysis of feeding behavior was conducted using the BioDAQ episodic food intake monitoring system for rats (BioDAQ, Research Diets, Inc., New Brunswick, NJ, USA) which allows the continuous monitoring of solid chow food intake in undisturbed rats as recently reported (Teuffel et al., 2015). The system contains a food hopper placed on an electronic microbalance, both are mounted on a regular rat single housing cage. Food intake parameters are measured continuously and can be extracted from the software (BioDAQ Monitoring Software 2.3.07); periods of interest can be chosen freely afterwards for data analysis. Every interaction of the rat with the food hopper is registered as a “bout.” A meal is defined as food intake of at least 0.01 g, when feeding bouts occur after an interval of ≥15 min this is considered a new meal. Meal parameters extracted from the software include bout size, meal size, bout frequency, meal frequency, meal duration, time spent in meals and eating rate. The food intake microstructure was analyzed starting at 4 days after food restriction over a period of 4 days (expressed as mean value of 4 days/animal).

### Monitoring of physical activity

Physical activity in the running wheel was assessed electronically using the software provided by the manufacturer (Campden Instruments Ltd., Loughborough, UK) and expressed as wheel rotations per day as described before (Wu et al., 2014). Here, the activity system was combined with the cages for automated food intake monitoring. Pilot studies did not indicate any deleterious interference between the two measurements (data not shown).

The estimation of energy consumption was based on an earlier study that determined oxygen consumption of rats running at a constant speed (Shepherd and Gollnick, 1976). A respiratory exchange ratio of 1.0 was assumed based on carbohydrates as largest component in the standard rat chow used (58% of calories from carbohydrates, manufacturer's information).

### c-Fos immunohistochemistry

At the end of the observation period, brain activation was assessed using c-Fos immunohistochemistry in *ad libitum* and activity-based anorexia rats (*n* = 3/group). In order to avoid signals from overfeeding and great distention of the stomach, food intake was restricted to 1.5 g in this last 1.5-h feeding period in the activity-based anorexia group. Directly after this feeding period, animals were perfused and brains processed for Fos immunohistochemistry as described before (Wang et al., 2011). Briefly, rats were deeply anesthetized by an intraperitoneal injection of 100 mg/kg ketamine (Ketanest™, Curamed, Karlsruhe, Germany) and 10 mg/kg xylazine (Rompun™2%, Bayer, Leverkusen, Germany). Transcardial perfusion was performed as described before (Stengel et al., 2009). After thoracotomy a cannula was inserted into the ascending aorta *via* the left heart ventricle. Perfusion consisted of a 1-min flush with sodium chloride (0.9%) followed by 500 ml of fixative (4% paraformaldehyde and 14% saturated picric acid in 0.1 M phosphate buffer, pH adjusted to 7.4). Afterwards, brains were removed and postfixed overnight in the same fixative at 4°C followed by a cryoprotection in 10% sucrose for 24 h. Lastly, brains were snap-frozen in dry ice-cooled 2-methylbutane (Carl Roth GmbH, Karlsruhe, Germany) and then stored at −80°C until further processing.

Rat brains from the two groups were processed in parallel to ensure similar conditions. Whole brains were cut into coronal sections (25 μm) from prefrontal forebrain to the caudal medulla using a cryostat (CryoStar NX70, Thermo Fisher Scientific, Waltham, MA, USA). Every third brain section was rinsed in phosphate-buffered saline (PBS) for 3 × 15 min. All incubations were performed using the free-floating technique at room temperature (except for the incubation with the primary antibody at 4°C) and followed by a 3 × 15 min washing step in PBS. The sections were first treated with 0.3% H_2_0_2_ in PBS for 30 min to block endogenous peroxidase activity. After rinsing the sections, nonspecific binding was blocked by 2% normal goat serum (NGS, Jackson ImmunoResearch Laboratories Inc., West Grove, PA, USA) for another 30 min. Sections were washed again and incubated in rabbit polyclonal anti-cFos (1:20,000, Catalog No. ABE457, Merck Millipore, Darmstadt, Germany) as primary antibody (2 h at room temperature followed by overnight at 4°C). Sections were rinsed again and incubated with biotinylated secondary goat anti-rabbit IgG (1:1000, Catalog No. 111-065-144, Jackson ImmunoResearch) for 2 h. After rinsing, this was followed by the incubation with the avidin-biotin-peroxidase complex (ABC, 1:500, Vector Laboratories, Burlingame, CA, USA) in 0.3% Triton-PBS for 1 h. Staining was visualized with diaminobenzidine tetrahydrochloride (DAB, Sigma-Aldrich, Darmstadt, Germany) and nickel ammonium sulfate (Fisher Scientific, Waltham, MA, USA). The color development was frequently checked with a light microscope and stopped after about 10 min. After staining, sections were mounted, air-dried, completely dehydrated through a gradient of ethanol, cleared in xylene and cover-slipped with Entellan™ new (Merck Millipore).

In a separate experiment, specificity of the cFos antibody was assessed by pre-absorption with synthetic SGFNADYEASSSRC (amino acids 4–17 of rat c-Fos, JPT Peptide Technologies GmbH, Berlin, Germany). The peptide (5 μg/ml) was incubated with the anti-c-Fos antibody diluted at 1:20,000 (Merck Millipore, antigen:antibody ratio of 100:1) for 2 h at room temperature followed by 22 h at 4°C. The solution was centrifuged for 15 min at 13,000 × g and the supernatant used for immunostaining as described above.

Immunoreactivity of brain sections was examined using a light microscope (Axiophot, Zeiss, Jena, Germany) and images were acquired using a connected camera (AxioCam HRc, Zeiss). The density of Fos positive cells in each brain section was determined semi-quantitatively using a 10x objective and described as −, no; +, low (~1–10 cells); ++, medium (~10–20 cells); and +++, high (>20 Fos positive cells in a 100 μm × 100 μm area of an ocular grid with a 10x objective) density of expression. Coordinates of the brain nuclei were identified according to the rat brain atlas (Paxinos and Watson, 2007). The investigator was blinded to the experimental group. The average density of Fos immunoreactive cells derived from the total number of sections analyzed for each nucleus was determined for each animal and used to calculate the mean density of expression per group.

### Statistical analysis

Distribution of the data was determined by the Kolmogorov-Smirnov test. Data are expressed as mean ± SEM and analyzed by one-way analysis of variance (ANOVA) followed by Tukey *post-hoc* test or two-way or three-way analysis of variance followed by the Holm-Sidak method. Differences were considered significant when *p* < 0.05 (SigmaStat 3.1., Systat Software, San Jose, CA, USA).

## Results

### Activity-based anorexia rats show the greatest reduction in body weight

**Figure 1:**
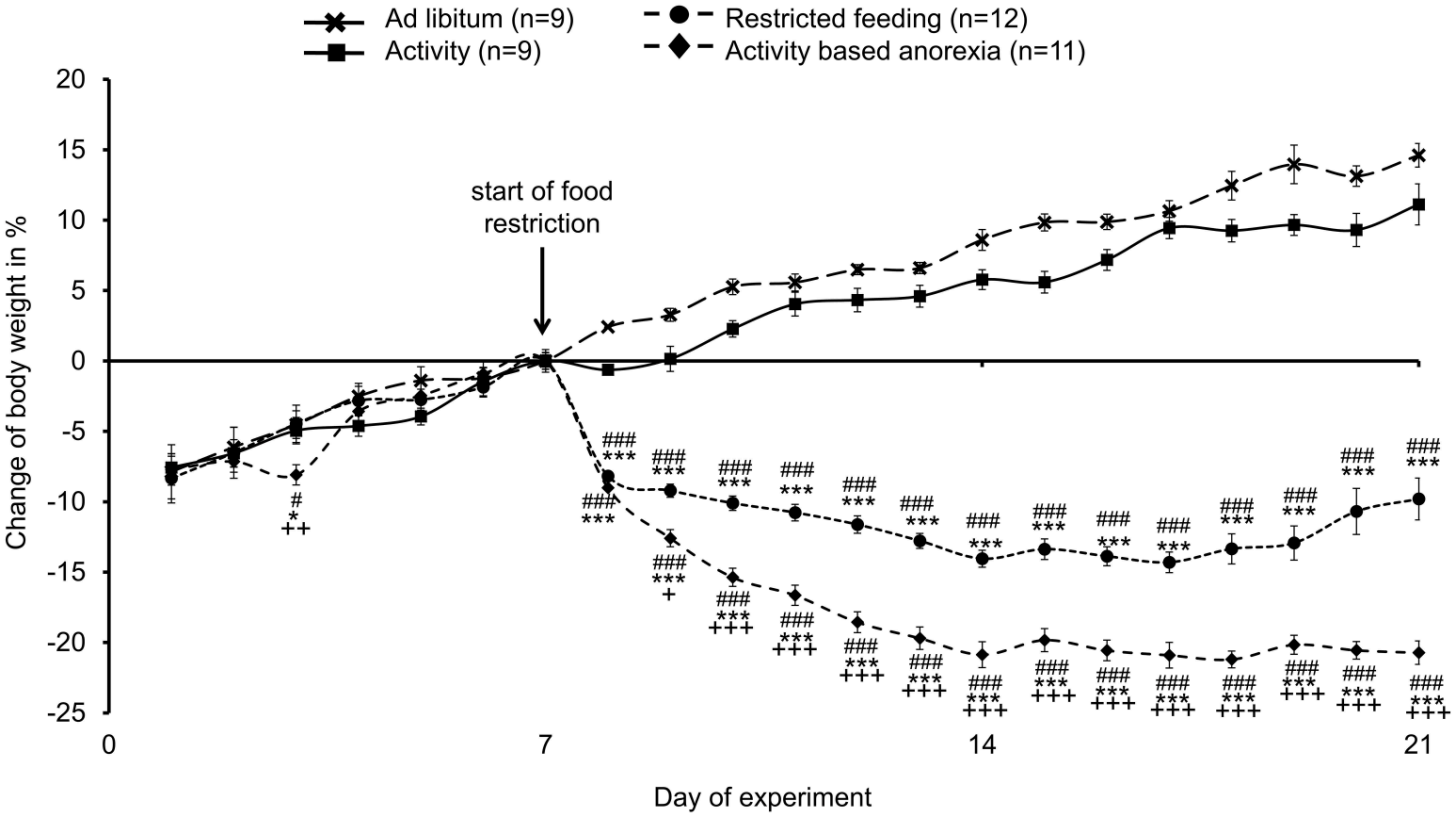
**Activity-based anorexia rats show the greatest body weight loss compared to all other groups**. Animals had access to a running wheel for 24 h/day (activity and activity-based anorexia group) or were housed without wheel access under otherwise similar conditions (*ad libitum* and restricted feeding group). On day eight food intake was restricted to 1.5 h/day in the restricted feeding and activity-based anorexia group, while the activity and *ad libitum* group retained access to food for 24 h/day. Body weight changes are expressed in % changes from the day of food restriction. Data are expressed as mean ± SEM. ^*^p < 0.05 and ^***^p < 0.001 vs. *ad libitum* group; ^#^*p* < 0.05 and ^*###*^*p* < 0.001 vs. activity group; +*p* < 0.05, ++*p* < 0.01 and +++*p* < 0.001 vs. restricted feeding group.

During the first week of the experiment (access to the running wheel in single housing cages for activity and activity-based anorexia group; regular single housing conditions for *ad libitum* and restricted feeding group) no body weight differences were observed between the four groups (Figure 1). After the start of food restriction, rats of the restricted feeding group as well as activity-based anorexia rats showed a body weight decrease, while the *ad libitum* and activity groups continued to gain body weight (Figure 1). At the end of the 14-day food restriction period, rats of the restricted feeding group showed a body weight decrease of −13% vs. day eight (*p* < 0.001) and vs. AC (−22%, *p* < 0.001) and AL (−26%, *p* < 0.001) that gained body weight (+10% and +13%, respectively; *p* < 0.001; Figure 1). Activity-based anorexia rats showed an additional body weight loss of −9% compared to rats of the restricted feeding group (*p* < 0.001; Figure 1) reaching an average body weight loss of −22% during the 14-day observation period. Three-way ANOVA showed a significant influence of time [*F*_(13, 504)_ = 5.6, *p* < 0.001], activity [*F*_(1, 504)_ = 436.1, *p* < 0.001] and feeding regimen [*F*_(1, 504)_ = 9806.3, *p* < 0.001] as well as an interaction of these three factors [*F*_(13, 504)_ = 2.0, *p* = 0.02].

It is to note that three out of 14 rats subjected to activity-based anorexia failed to lose body weight (or even gained body weight during the experimental period, data not shown) and were therefore excluded from further analyses (final *n* = 11).

### Activity-based anorexia rats show a similar reduction in food intake as observed in the restricted feeding group

**Figure 2:**
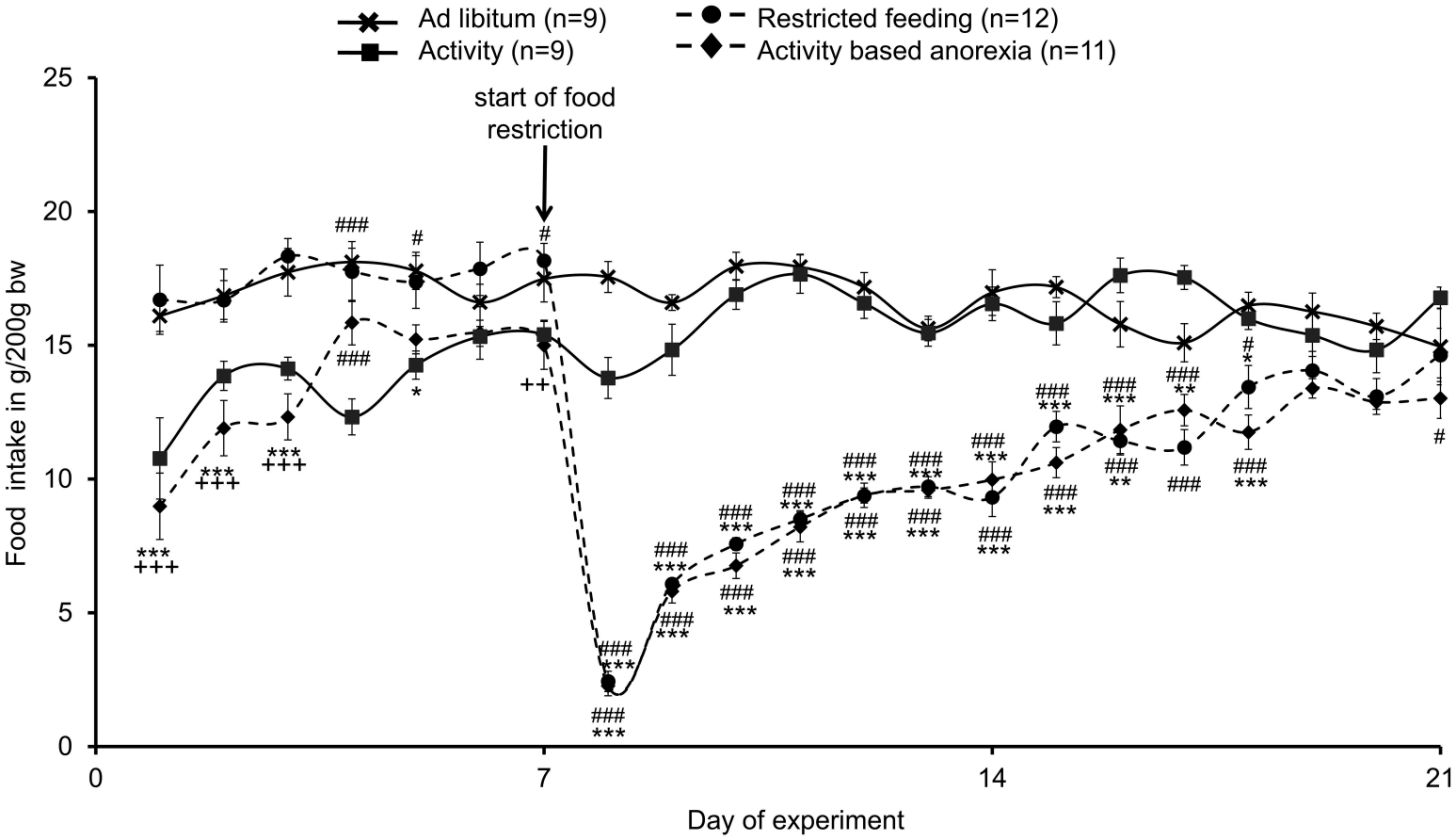
**Activity-based anorexia and restricted feeding group show a similar reduction in food intake over the whole observation period**. Animals had access to a running wheel for 24 h/day (activity and activity-based anorexia group) or were housed without wheel access under otherwise similar conditions (*ad libitum* and restricted feeding group). On day eight food intake was restricted to 1.5 h/day in the restricted feeding and activity-based anorexia group, while the activity and *ad libitum* group retained access to food for 24 h/day. Food intake is calculated as g/200 g body weight; all data are expressed as mean ± SEM. ^*^p < 0.05, ^**^p < 0.01, and ^***^p < 0.001 vs. *ad libitum* group; ^#^*p* < 0.05 and ^*###*^*p* < 0.001 vs. activity group; ++*p* < 0.01 and +++*p* < 0.001 vs. restricted feeding group.

**Figure 3:**
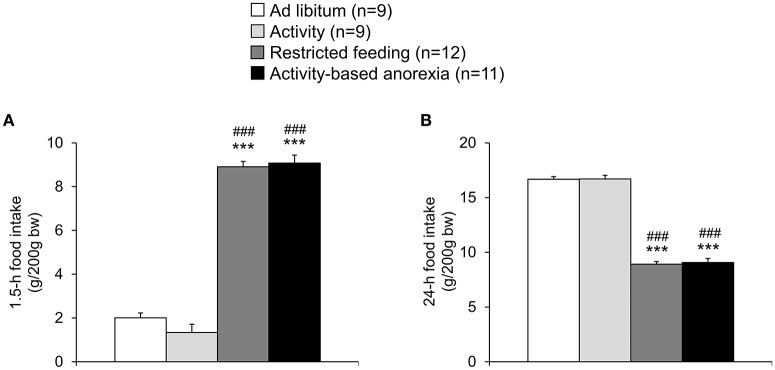
**Activity-based anorexia and restricted feeding group show a similar reduction in 1.5-h and 24-h food intake**. Animals had access to a running wheel for 24 h/day (activity and activity-based anorexia group) or were housed without wheel access under otherwise similar conditions (*ad libitum* and restricted feeding group). On day eight food intake was restricted to 1.5 h/day in the restricted feeding and activity-based anorexia group, while the activity and *ad libitum* group retained access to food for 24 h/day. Food intake was monitored continuously using an automated food intake monitoring device and 1.5-h **(A)** as well as 24-h **(B)** food intake analyzed over a period of 4 days starting 4 days after food restriction (when a relatively stable food intake was observed). Data are expressed as mean ± SEM. ^***^p < 0.001 vs. *ad libitum* group and ^*###*^*p* < 0.001 vs. activity group.

The food intake observed in the two *ad libitum* fed groups (*ad libitum* and activity group) did not differ from each other and was fairly stable over the 21-day observation period (Figure 2). While during the first 3 days of the habituation period—although during this time also fed *ad libitum*—the food intake was lower in the activity as well as activity-based anorexia group compared to the *ad libitum* and the restricted feeding groups (*p* < 0.001) giving rise to more time spent for physical activity and less for food intake, food intake was similar on days 6 and 7 (before food restriction) in all four groups (Figure 2). After food restriction to 1.5 h per day in the restricted feeding and activity-based anorexia group, food intake significantly dropped by −88% in these groups on the first day compared to the *ad libitum* group (*p* < 0.001) and slowly increased afterwards to reach the same level as observed in the *ad libitum* fed groups on the last day of the observation period (Figure 2). Overall, rats of the restricted feeding and activity-based anorexia group ate less (−38 and −41%, respectively) compared to the *ad libitum* group during the 14-day food restriction period (Figure 2). Three-way ANOVA indicated a significant impact of time [*F*_(20, 776)_ = 24.5, *p* < 0.001], activity [*F*_(1, 776)_ = 68.3, *p* < 0.001] and feeding regimen [*F*_(1, 776)_ = 626.5, *p* < 0.001] as well as an interaction of these three factors [*F*_(20, 776)_ = 1.8, *p* = 0.02]. Food intake during 1.5 h did not differ between the restricted feeding and activity-based anorexia group, while 24-h food intake did not differ between the *ad libitum* and activity group (*p* > 0.05; Figure 3).

### Activity-based anorexia rats show a similar physical activity as observed in the activity group

**Figure 4:**
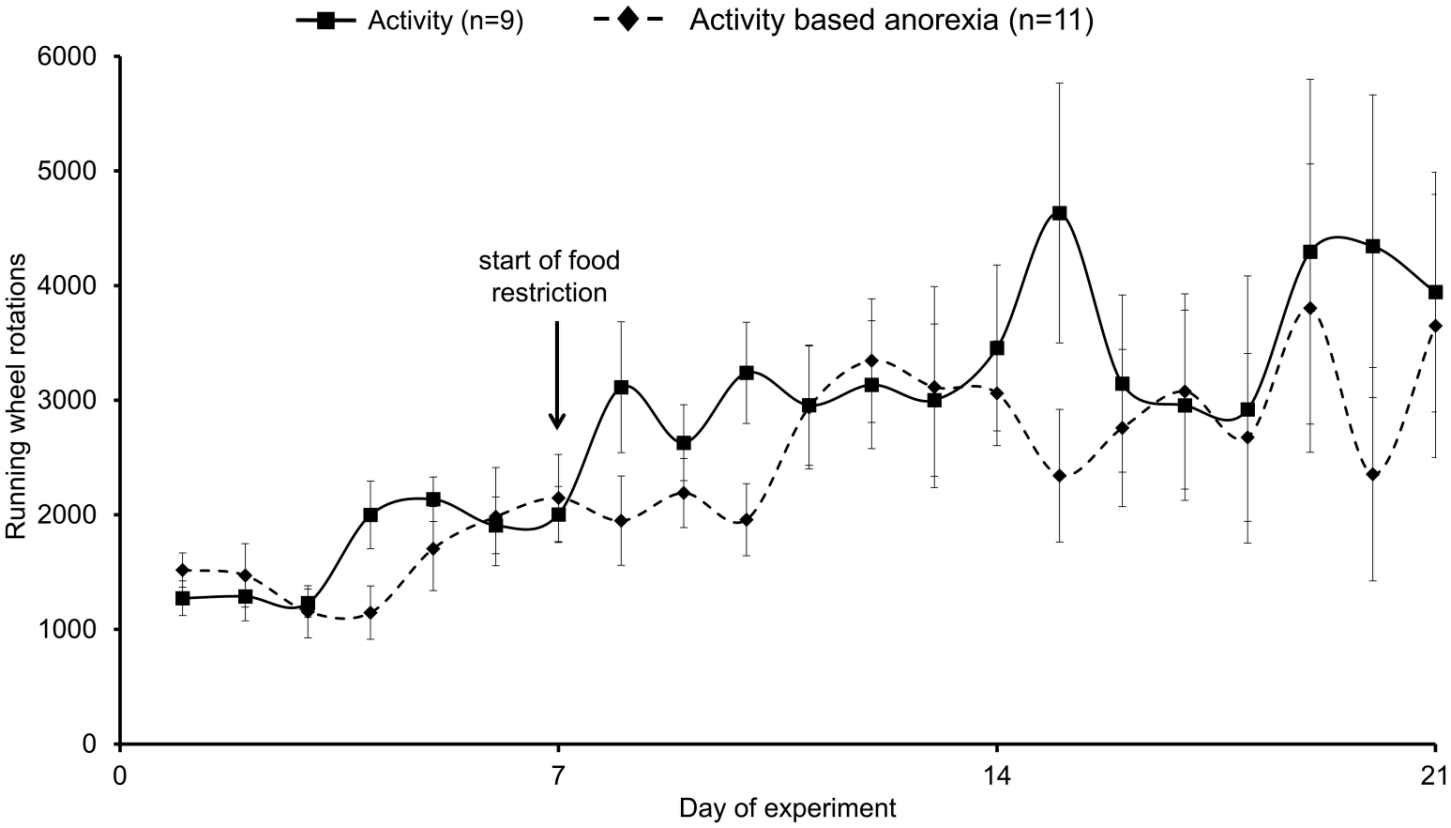
**Activity-based anorexia and activity group fed *ad libitum* show similar levels of physical activity**. Animals had access to a running wheel for 24 h/day. On day eight food intake was restricted to 1.5 h/day in the activity-based anorexia group, while the activity group retained access to food for 24 h/day. Physical activity was monitored by measuring the wheel rotations per day. Data are expressed as mean ± SEM. *p* > 0.05.

Physical activity assessed using a running wheel slightly increased during the first week from ~1500 to ~2000 wheel rotations/day (Figure 4). During the food restriction period, physical activity more prominently increased reaching ~3500 wheel rotations/day in both, the activity and activity-based anorexia groups (Figure 4), corresponding to ~1300 m/day. No daily differences were observed either in the 1.5-h (data not shown) or 24-h wheel rotations between the activity and activity-based anorexia group (*p* > 0.05; Figure 4).

The daily energy expenditure including calculated resting energy expenditure and energy expenditure while running was ~35 kcal/200 g body weight at the beginning of the observation period (data not shown). This value slightly increased during the food restriction period in both the activity and activity-based anorexia group to ~38 kcal/200 g body weight (data not shown). Two-way ANOVA indicated a significant impact of time [*F*_(20, 377)_ = 3.6, *p* < 0.001] and feeding regimen [*F*_(1, 377)_ = 4.3, *p* = 0.04].

Caloric deficit was calculated by subtracting energy expenditure from calculated caloric intake. All four groups showed a caloric surplus during the first 7 days of the experimental period (ranging from 9.6 to 21.4 kcal/day on day 7, data not shown). While this surplus remained visible in the *ad libitum* fed groups over the remaining 14-day observation period (14.2 ± 4.6 kcal in the *ad libitum* and 17.1 ± 2.3 kcal in the activity group), in the restricted feeding groups a caloric deficit was observed from the start of the food restriction (greatest levels on day 8: restricted feeding: −30.5 ± 1.4 kcal, activity-based anorexia: −29.6 ± 0.9 kcal) with a progressive decrease of caloric deficit reaching a surplus again on day 21 (restricted feeding: 10.4 ± 3.6 kcal, activity-based anorexia: 2.0 ± 0.9 kcal, data not shown). Three-way ANOVA indicated a significant impact of time [*F*_(20, 771)_ = 22.3, *p* < 0.001], activity [*F*_(1, 771)_ = 125.8, *p* < 0.001] and feeding regimen [*F*_(1, 771)_ = 666.7, *p* < 0.001] as well as an interaction of these three factors [(*F*_(20, 771)_ = 2.0, *p* < 0.01].

### Activity-based anorexia rats show a similar food intake microstructure compared to the restricted feeding group

**Figure 5:**
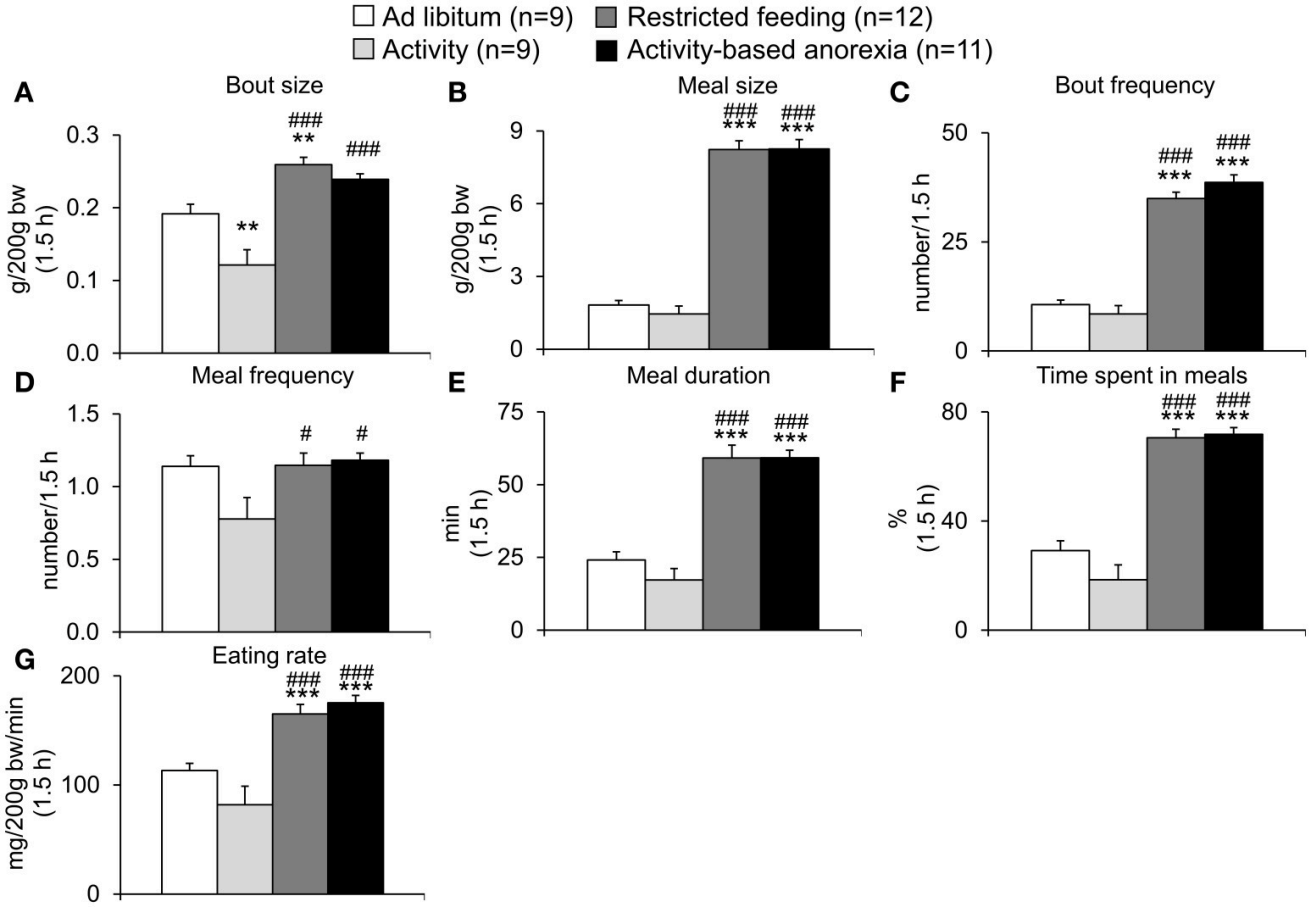
**Activity-based anorexia and restricted feeding group show similar changes in the 1.5-h food intake microstructure**. Animals had access to a running wheel for 24 h/day (activity and activity-based anorexia group) or were housed without wheel access under otherwise similar conditions (*ad libitum* and restricted feeding group). On day eight food intake was restricted to 1.5 h/day (from 9:00 to 10:30 a.m.) in the restricted feeding and activity-based anorexia group, while the activity and *ad libitum* group retained access to food for 24 h/day. Food intake was monitored continuously using an automated food intake monitoring device and parameters of the food intake microstructure during the 1.5-h restricted feeding period, namely bout size **(A)**, meal size **(B)**, bout frequency **(C)**, meal frequency **(D)**, meal duration **(E)**, time spent in meals **(F)**, and eating rate **(G)** analyzed over a period of 4 days starting 4 days after food restriction (when a relatively stable food intake was observed). Data are expressed as mean ± SEM. ^**^p < 0.01 and ^***^p < 0.001 vs. *ad libitum* group; ^#^*p* < 0.05 and ^*###*^*p* < 0.001 vs. activity group.

After analysis of overall daily 24-h (in the *ad libitum* fed groups) and 1.5-h (in the restricted feeding groups) food intake, the underlying food intake microstructure was assessed using an automated food intake monitoring device. When analyzing the 1.5-h food intake microstructure no difference was observed between both restricted feeding groups (restricted feeding and activity-based anorexia, *p* > 0.05; Figures 5A–G). Similarly, the two *ad libitum* fed groups did not show a difference except for the bout size which was smaller in the activity compared to the *ad libitum* group (*p* < 0.01; Figure 5A). The two restricted feeding groups showed significantly higher levels for several parameters of the food intake microstructure such as meal size (*p* < 0.001; Figure 5B), bout frequency (*p* < 0.001; Figure 5C), meal duration (*p* < 0.001; Figure 5E), time spent in meals (*p* < 0.001; Figure 5F) and eating rate (*p* < 0.001; Figure 5G), while for meal frequency the difference was only observed when compared to the activity group (*p* < 0.05; Figure 5D).

**Figure 6:**
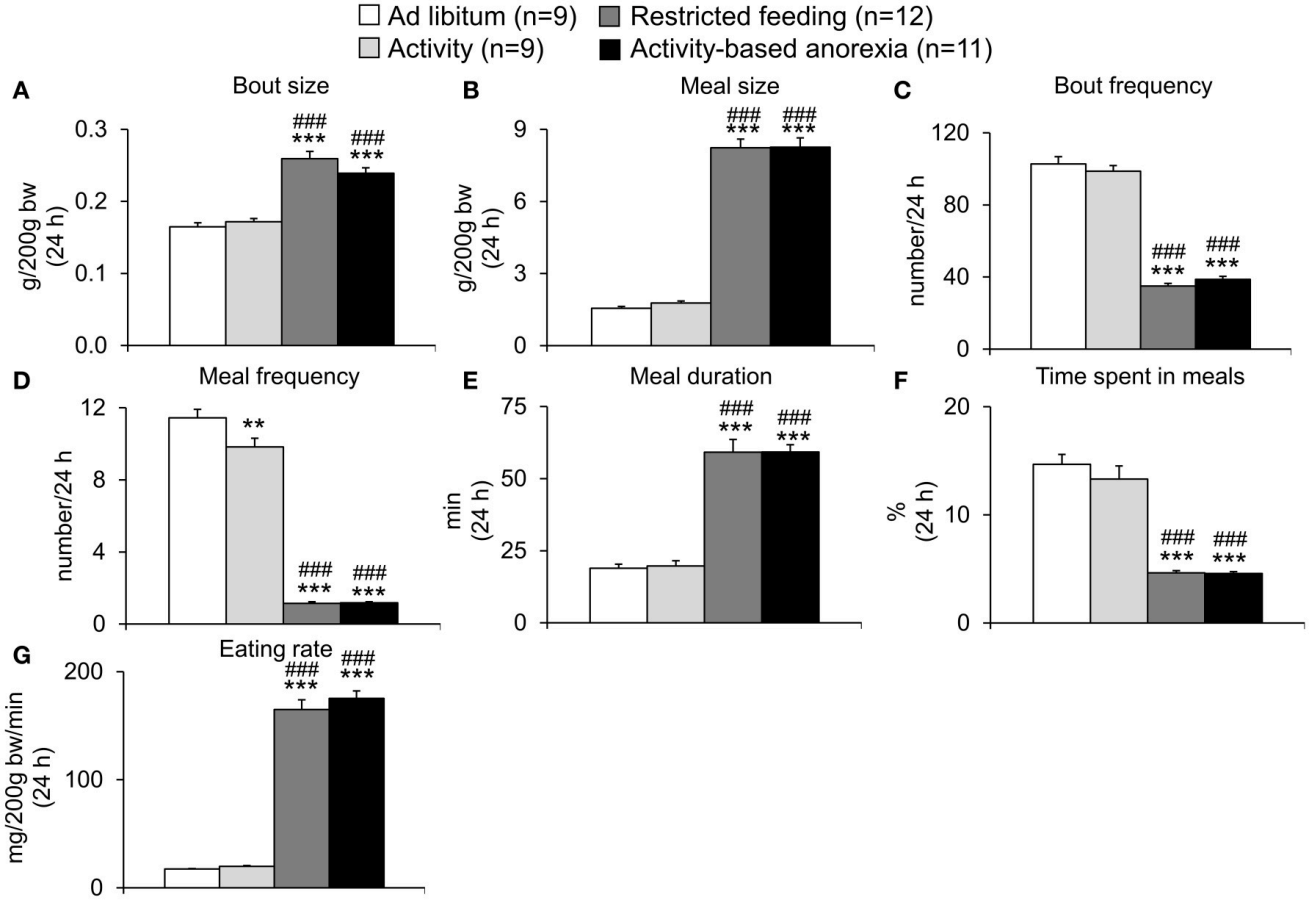
**Activity-based anorexia and restricted feeding group show similar changes in the 24-h food intake microstructure**. Animals had access to a running wheel for 24 h/day (activity and activity-based anorexia group) or were housed without wheel access under otherwise similar conditions (*ad libitum* and restricted feeding group). On day eight food intake was restricted to 1.5 h/day (from 9:00 to 10:30 a.m.) in the restricted feeding and activity-based anorexia group, while the activity and *ad libitum* group retained access to food for 24 h/day. Food intake was monitored continuously using an automated food intake monitoring device and parameters of the 24-h food intake microstructure, namely bout size **(A)**, meal size **(B)**, bout frequency **(C)**, meal frequency **(D)**, meal duration **(E)**, time spent in meals **(F)**, and eating rate **(G)** analyzed over a period of 4 days starting 4 days after food restriction (when a relatively stable food intake was observed). Data are expressed as mean ± SEM. ^**^p < 0.01 and ^***^p < 0.001 vs. *ad libitum* group and ^*###*^*p* < 0.001 vs. activity group.

Also when analyzing the 24-h food intake microstructure, no difference was observed between the two *ad libitum* fed groups (*ad libitum* and activity) or the two restricted feeding groups (restricted feeding and activity-based anorexia, *p* > 0.05; Figures 6A–G). While both restricted feeding groups showed higher levels in bout size (*p* < 0.001; Figure 6A), meal size (*p* < 0.001; Figure 6B), meal duration (*p* < 0.001; Figure 6E) and eating rate (*p* < 0.001; Figure 6G), values were lower for—as expected based on the feeding schedule—bout frequency (*p* < 0.001; Figure 6C), meal frequency (*p* < 0.001; Figure 6D) and time spent in meals (*p* < 0.001; Figure 6F).

### Activity-based anorexia robustly activates several brain nuclei in different areas of the brain

To investigate neuronal activation of brain areas under conditions of activity-based anorexia we performed immunohistochemistry for the activity marker Fos. After pre-absorption of the Fos antibody with a synthetic Fos fragment no immunostaining was observed (data not shown) indicating the specificity of the antibody.

**Figure 7:**
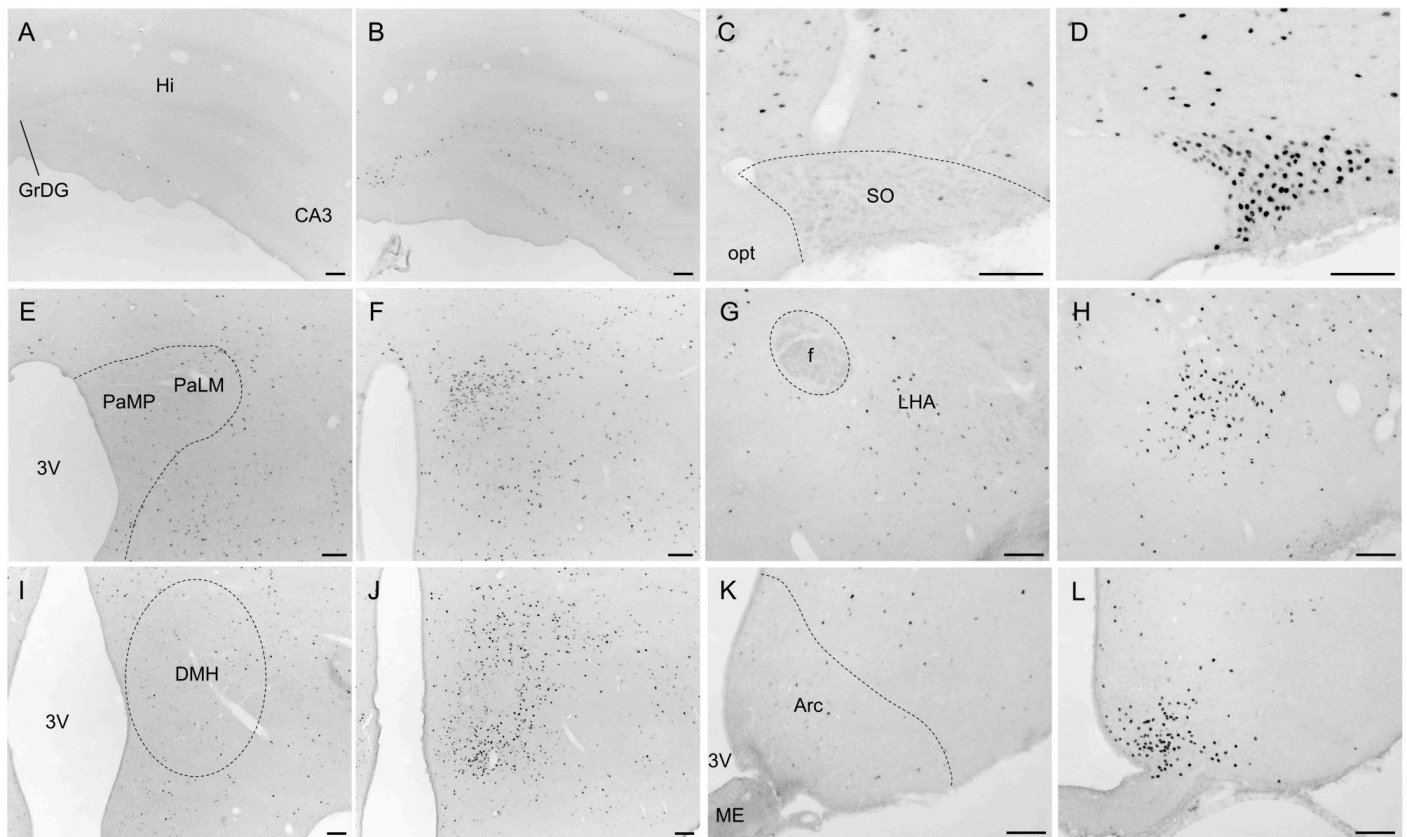
**Representative microphotographs of forebrain and hypothalamic structures in rats under *ad libitum* or activity-based anorexia conditions**. Following the 21-day observation period rats received food for 1.5 h (activity-based anorexia) or were fed *ad libitum* and were transcardially perfused directly after this 1.5-h feeding period. It is to note that rats in the activity-based anorexia group received a fixed amount of 1.5 g during this feeding period in order to avoid signals derived from overfilling of the stomach and/or nausea. Brains were processed for Fos immunohistochemistry. Signals were observed in the hippocampus **(B)**, supraoptic nucleus **(D)**, magno- und parvocellular parts of the paraventricular nucleus **(F)**, lateral hypothalamic area **(H)**, dorsomedial hypothalamic nucleus **(J)** and medial part of the arcuate nucleus **(L)** of activity-based anorexia rats, while in the respective nuclei of the *ad libitum* group no **(C,I)** or few **(A,E,G,K)** signals were detected. The scale bars indicate 100 μm. Abbreviations: 3V, third ventricle; Arc, arcuate nucleus; CA3, field CA3 of the hippocampus; DMH, dorsomedial hypothalamic nucleus; f, fornix; GrDG, granular layer of the dentate gyrus; Hi, hippocampus; LHA, lateral hypothalamic area; ME, median eminence; opt, optic tract; PaLM, lateral magnocellular part of the paraventricular nucleus of the hypothalamus; PaMP, medial parvocellular part of the paraventricular nucleus of the hypothalamus; SO, supraoptic nucleus.

**Figure 8:**
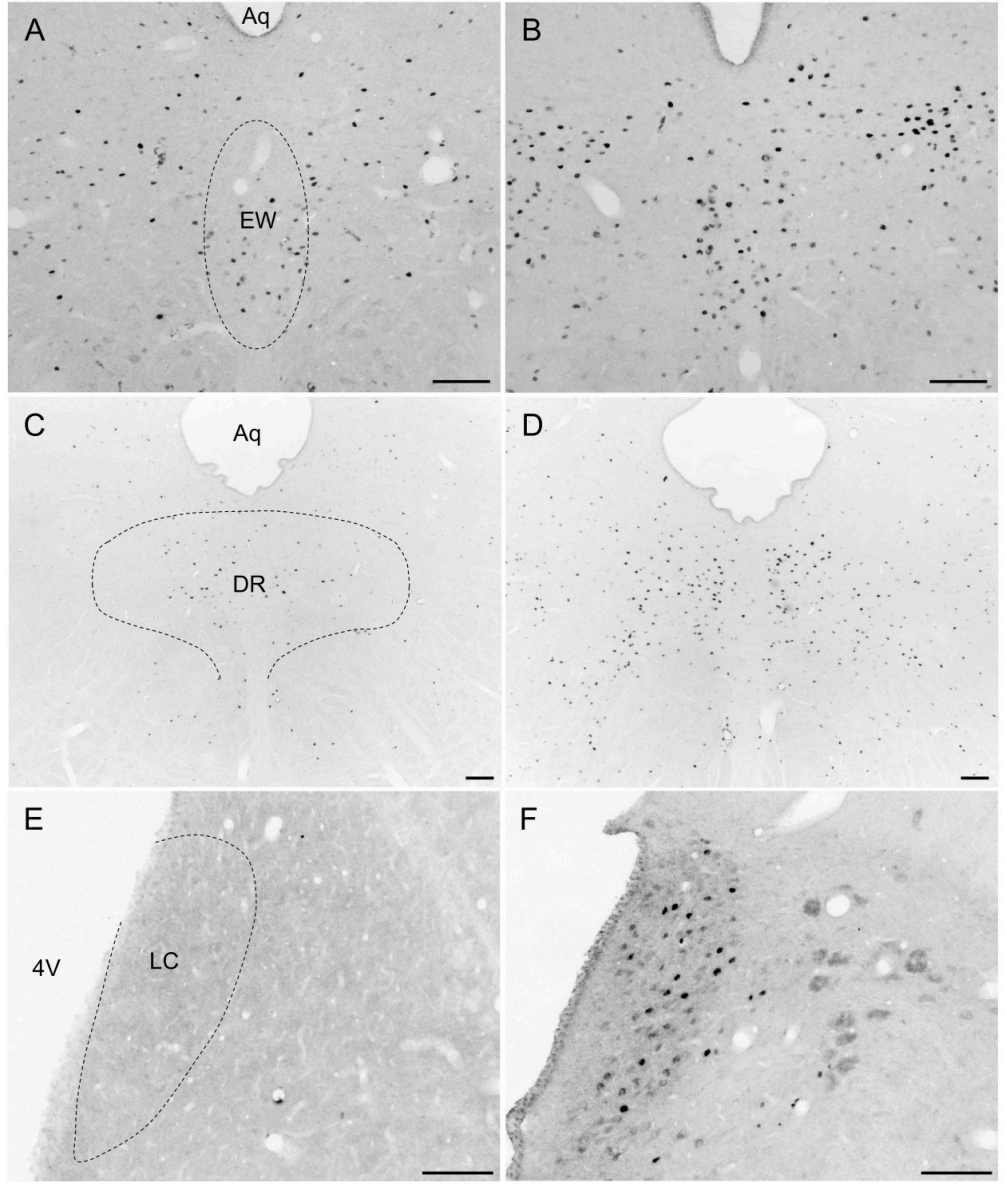
**Representative microphotographs of midbrain structures in rats under *ad libitum* or activity-based anorexia conditions**. Following the 21-day observation period rats received food for 1.5 h (activity-based anorexia) or were fed *ad libitum* and were transcardially perfused directly after this 1.5-h feeding period. It is to note that rats in the activity-based anorexia group received a fixed amount of 1.5 g during this feeding period in order to avoid signals derived from overfilling of the stomach and/or nausea. Brains were processed for Fos immunohistochemistry. Signals were observed in the Edinger-Westphal nucleus **(B)**, dorsal raphe nuclei **(D)** and locus coeruleus **(F)** of the activity-based anorexia group, while in the respective nuclei of the *ad libitum* group no **(E)**, or few **(C)** signals were detected. It is to note that signals of similar density were observed in the Edinger-Westphal nucleus **(A)** of the *ad libitum* group. The scale bars indicate 100 μm. Abbreviations: 4V, fourth ventricle; Aq, aqueduct; DR, dorsal raphe nuclei; EW, Edinger-Westphal nucleus; LC, locus coeruleus.

**Figure 9:**
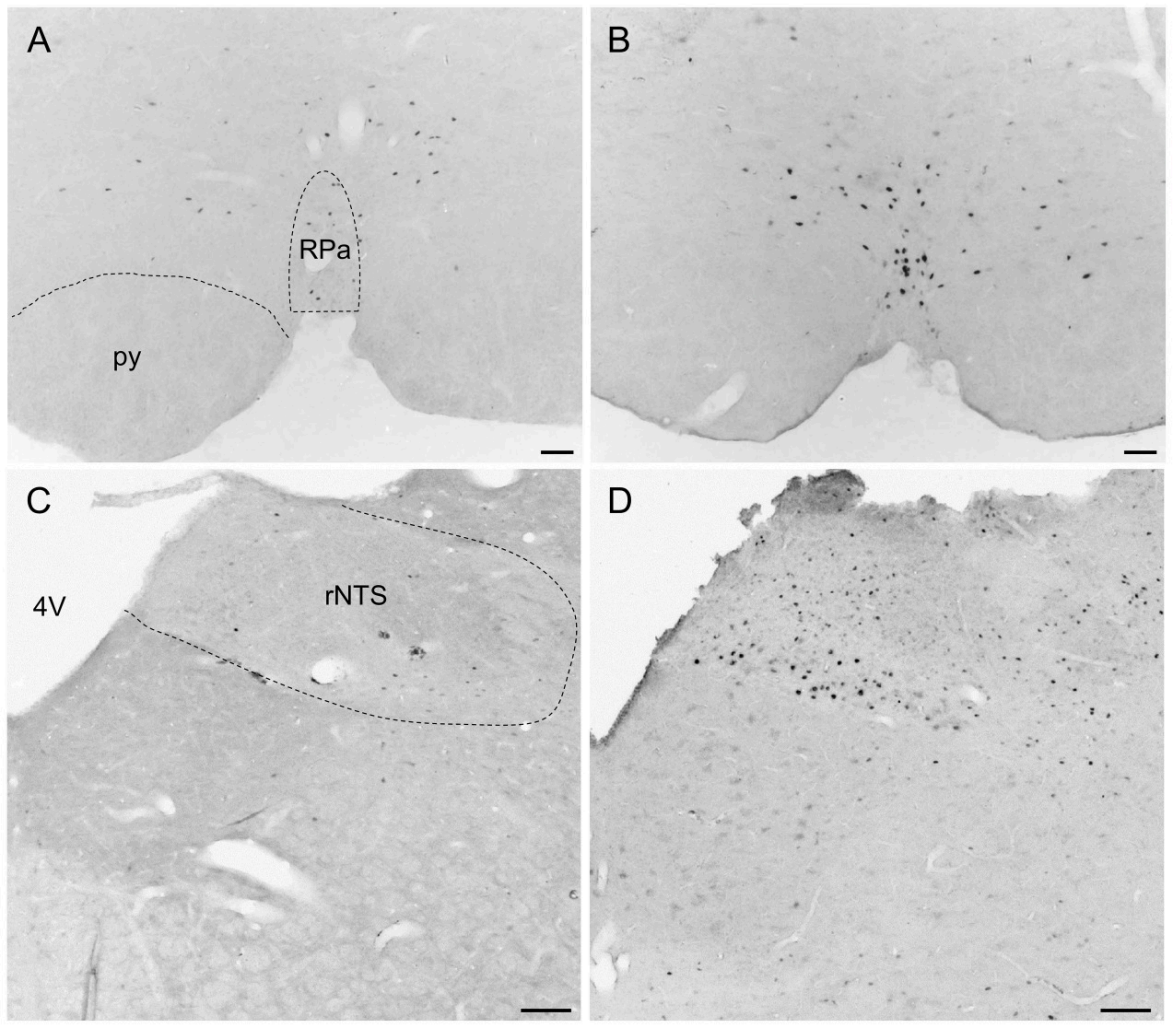
**Representative microphotographs of medulla structures in rats under *ad libitum* or activity-based anorexia conditions**. Following the 21-day observation period rats received food for 1.5 h (activity-based anorexia) or were fed *ad libitum* and were transcardially perfused directly after this 1.5-h feeding period. It is to note that rats in the activity-based anorexia group received a fixed amount of 1.5 g during this feeding period to avoid signals derived from overfilling of the stomach and/or nausea. Signals were observed in the raphe pallidus nucleus **(B)** and the rostral part of the nucleus of the solitary tract **(D)** of the activity-based anorexia group, while in the respective nuclei of the *ad libitum* group no **(C)** or few **(A)** Fos positive neurons were detected. The scale bars indicate 100 μm. Abbreviations: 4V, fourth ventricle; py, pyramidal tract; rNTS, rostral part of the nucleus of the solitary tract; RPa, raphe pallidus nucleus.

Overall, activity-based anorexia rats showed higher Fos activation levels compared to *ad libitum* fed rats (Table 1). In the forebrain, higher activity was observed in the piriform cortex, cingulate cortex, somatomotor cortex, lateral septal nucleus, caudate putamen and hippocampus (Figures 7A,B) of activity-based anorexia rats compared to the *ad libitum* group, while no activation was observed in the amygdala (Table 1). In the thalamus higher Fos activation was observed in the paraventricular thalamic nucleus of activity-based anorexia rats compared to the *ad libitum* group, while in the lateral habenula similar numbers of Fos positive cells were detected using semiquantitative assessment (Table 1). Evaluation of hypothalamic nuclei showed more Fos signals in the suprachiasmatic nucleus, supraoptic nucleus (Figures 7C,D), anterior hypothalamic area, both magno- and parvocellular parts of the paraventricular nucleus (Figures 7E,F), lateral hypothalamic area (Figures 7G,H), dorsomedial hypothalamic nucleus (Figures 7I,J) and the medial part of the arcuate nucleus (Figures 7K,L) of activity-based anorexia rats compared to the *ad libitum* group, while similar levels were observed in the ventromedial hypothalamic nucleus (Table 1). In the midbrain similar levels were detected in the Edinger-Westphal nucleus (Figures 8A,B), while higher activation was observed in the dorsal raphe nuclei (Figures 8C,D) and locus coeruleus (Figures 8E,F) of activity-based anorexia compared to the *ad libitum* fed rats (Table 1). Lastly, also in the medulla a higher activation was observed in activity-based anorexia rats compared to the *ad libitum* group, namely in the raphe pallidus nucleus (Figures 9A,B), area postrema, rostral part of the nucleus of the solitary tract (Figures 9C,D) and the dorsal motor nucleus of the vagus nerve (Table 1).

**Table 1:** **Localization of Fos positive neurons in brains of rats fed *ad libitum* or under conditions of activity-based anorexia**.

**Area**	**Brain structure**	***Ad libitum***	**Activity based anorexia**
Forebrain	Piriform cortex	++	+++
	Cingulate cortex	+	++
	Somatomotor cortex	+	+++
	Lateral septal nucleus	+ −++	+++
	Caudate putamen	++	++ −+++
	Amygdala (central, medial and basolateral)	−	−
	Hippocampus	+ −++	+++
Thalamus	Paraventricular thalamic nucleus, anterior part	+	++
	Lateral habenula	+ −++	+ −++
Hypothalamus	Suprachiasmatic nucleus	++	++ −+++
	Supraoptic nucleus	−	+++
	Anterior hypothalamic area	+	++
	Paraventricular nucleus	+	++
	Lateral hypothalamic area	++	+++
	Ventromedial hypothalamic nucleus	++	++
	Dorsomedial hypothalamic nucleus	−	++
	Arcuate nucleus, medial	+	+++
Midbrain	Edinger-Westphal nucleus	++	++
	Dorsal raphe nuclei	+	++
	Locus coeruleus	−	+
Medulla	Raphe pallidus nucleus	+	++
	Area postrema	−	+ −++
	Nucleus of the solitary tract, rostral	−	+ −++
	Dorsal motor nucleus of the vagus nerve	−	−−+

*Data are expressed as mean derived from three rats/group. It is to note that rats in the activity-based anorexia group received a fixed amount of 1.5 g during the feeding period in order to avoid signals derived from overfilling of the stomach and/or nausea. Fos expression is expressed semi-quantitatively as +, low (~1–10 cells); ++, medium (~10–20 cells); and +++, high (>20 Fos positive cells in a 100 μm × 100 μm area of an ocular grid with a 10x objective) density of expression; −, no cells*.

## Discussion

In the present study we first established the activity-based anorexia model combining voluntary physical activity in a running wheel and a time-restricted feeding protocol and showed a body weight loss of −22% following 2 weeks of restricted feeding for 1.5 h per day and 24-h access to a running wheel. This body weight loss was greater (−9%) than that observed in the restricted feeding group, while rats of the *ad libitum* and activity group gained body weight. These data indicate the importance of food restriction for the present model. To gain further insight into the underlying changes, we next analyzed the food intake microstructure which greatly differed from the microstructure observed in the *ad libitum* fed groups (*ad libitum* and activity group) but was very similar to the one observed in the restricted feeding group. The increase in 1.5-h food intake observed in the two groups kept on the restricted feeding schedule was based on a larger meal size and an increase in eating rate. During these 1.5 h, rats spent 71% of the time in meals (eating and interacting with the food hopper). It is important to note that despite this great drive to eat, rats still exercised during this 1.5-h period.

Interestingly, daily activity was similar between the activity and activity-based anorexia group; however, daily activity increased over time in both groups. These data are in line with previous data from mice where daily activity also increased over time; however, in mice daily activity decreased during the last days of the 2.5-week observation period (Jésus et al., 2014). Whether this represents a species difference (rats vs. mice), sex difference (female vs. male) or is related to the slower weight loss in rats (−20% reached at day 15 vs. 11 in mice) warrants further investigation. At the same time we did not observe a decrease in food intake (which rather plateaued) which is different from the human situation where food intake decreases while activity increases with the progression of the disease (Davis et al., 1994). The relatively short observation period of 3 weeks likely contributes to/explains this difference. Taken together, the combination of both food restriction and activity is key in order to exert the pronounced weight loss observed in the activity-based anorexia group.

It is to note that in the present study three out of 14 rats did not develop activity-based anorexia and were therefore excluded from further analyses. This finding is in line with previous studies reporting that 20–30% of rats are not interested in running (Mondon et al., 1985) and do not develop activity-based anorexia (Carrera et al., 2014). These data well match the dropout rate described here (3 out of 14 = 21%). Interestingly, this finding also parallels human data where up to 80% of anorexic subjects display hyperactivity, whereas 20% do not (Davis et al., 1997). Whether a difference in leptin levels, hypothesized to play a role in the semi-starvation-induced hyperactivity in rats before (Exner et al., 2000), contributes to these differences will have to be further investigated. On the other hand, all rats of the restricted feeding group (12/12) show a reduction in body weight indicating that this effect cannot be eluded. However, the fact that rats of the activity-based anorexia group show a greater body weight loss than the restricted feeding group highlights the importance of the physical activity.

Despite the fact that the data mentioned above give rise to the use of the activity-based anorexia model as a suited tool to study pathophysiological alterations of AN, several limitations should be kept in mind. Although restriction of food and increased physical activity, two mean features of AN (Treasure et al., 2015) are used in this model, several other aspects such as genetic susceptibility (Clarke et al., 2012) or psychosocial and interpersonal factors (Zipfel et al., 2015) are not respected. Moreover, rats do not voluntarily reduce their body weight in contrast to human anorexic subjects. Whenever the rats' access to food is increased again, they start to regain body weight (Dixon et al., 2003; Ratnovsky and Neuman, 2011). Furthermore, the changes induced here are rather acute or subacute, while human AN is a chronic disease. Interestingly, after the initial sharp decline of food intake rats of the activity-based anorexia and restricted feeding group show a gradual increase of food intake reaching similar levels of daily food intake as observed in the *ad libitum* fed groups. Whether different dietary patterns as observed in human anorexia (Huse and Lucas, 1984; Elran-Barak et al., 2014) or a change of dietary patterns over time occurs in these rats as well will have to be further determined, preferably in a study with a longer monitoring time. Taken together, cautious interpretation of the data obtained in this model is necessary.

Several other anorexia models have been developed encompassing genetically engineered mouse models that share similarities with changes observed in AN; however, none of these reflect the multiple hormonal changes observed in AN (Méquinion et al., 2015). Lastly, other models use access to low caloric food or expose rats to various kinds of stressors (Méquinion et al., 2015). However, it is important to note that so far activity-based anorexia is considered the best animal model (Gutierrez, 2013) as it recapitulates two main features, physical activity and reduced food intake, of human AN.

To further characterize the activity-based anorexia rats we also investigated the activation of brain nuclei using the activity marker Fos (Sagar et al., 1988) and performed a whole brain mapping for activity-based anorexia and *ad libitum* fed rats. Neuronal activation was observed in brain areas involved in the regulation of several functions such as motor activity, stress response, food intake and thermogenesis.

Analyzing brain areas involved in olfaction and the processing of olfactory stimuli (Roullet et al., 2005) an increased activation of neurons was observed in the piriform cortex, while in the lateral habenula similar Fos expression was observed in activity-based anorexia and *ad libitum* fed rats. This activation is likely associated with the increased interaction with food (as reflected in the increased number of bouts in activity-based anorexia rats compared to the *ad libitum* group) as well as the stimulated food intake during the 1.5-h feeding period. In line with this assumption, key areas of food intake regulation were activated as well, namely the lateral septal nucleus (Mitra et al., 2015), lateral hypothalamic area (Bernardis and Bellinger, 1993), the dorsomedial hypothalamic nucleus and the medial part of the Arc, both expressing the potent orexigenic transmitter neuropeptide Y (Wang et al., 2002; Bi et al., 2012) and lastly also the nucleus of the solitary tract (Stengel and Taché, 2011). Further corroborating the involvement of these nuclei in the orexigenic drive under conditions of activity-based anorexia, a previous study reported a robust upregulation of orexigenic agouti-related peptide and neuropeptide Y, whereas the anorexigenic transmitters pro-opiomelanocortin (POMC) and cocaine- and amphetamine-regulated transcript (CART) were reduced in the Arc of activity-based anorexia rats compared to sedentary food-restricted controls (de Rijke et al., 2005). Moreover, in the lateral hypothalamic area melanin-concentrating hormone mRNA expression was increased in activity-based anorexia rats (de Rijke et al., 2005). Associated with the orexigenic response, also brain nuclei involved in the regulation of gastrointestinal motility were activated, namely the lateral hypothalamic area (Gong et al., 2013), nucleus of the solitary tract and the dorsal motor nucleus of the vagus nerve (Stengel and Taché, 2011). This pronounced activation likely underlies the robust orexigenic response of activity-based anorexia rats observed during the 1.5-h feeding period. It is to note that—although food intake was restricted to 1.5 g in the last feeding period before brain processing for Fos immunohistochemistry to avoid unspecific gastric distention and nauseating signals—a moderate activation of the area postrema, known to be involved in the mediation of nausea (Horn, 2014), has been observed in the activity-based anorexia but not in the *ad libitum* fed group. Therefore, nauseating signals—at least to a certain extent—might play a role in this model as well and may modulate/limit food intake displayed during the restricted feeding period.

Besides the restriction of food intake, the stimulation of activity contributes to the weight loss observed in activity-based anorexia rats. Respective nuclei activated under these conditions and therefore likely implicated in the stimulation of activity encompass the somatomotor cortex (Elias et al., 2008) and caudate putamen (David et al., 2005). Interestingly, especially the dorsomedial hypothalamic nucleus has been implicated in the mediation of food-anticipatory activity under fixed-feeding conditions (Verhagen et al., 2011) as also observed in the present study. This activation likely also involves the suprachiasmatic nucleus working in a modulatory manner as part of an intrahypothalamic system (Acosta-Galvan et al., 2011). Moreover, the dorsomedial hypothalamic nucleus was implicated in the food-entrainable-related preprandial rise of body temperature, an effect that vanished after lesion of the nucleus (Gooley et al., 2006). This thermogenic response might also contribute to the observed decrease in body weight. However, it is important to note that activity-based anorexia was associated with a hypothermic response before (Hillebrand et al., 2005a) and an increase in ambient temperature was reported to reduce physical activity (Gutierrez et al., 2008). Future studies should further investigate these—likely very dynamic—changes of body temperature in activity-based anorexia rats.

Also stress mediated *via* the hypothalamic-pituitary-adrenal gland axis might play a role in the reduction of food intake and stimulation of physical activity. In the present study we observed a robust activation of lateral parvocellular neurons of the hypothalamic paraventricular nucleus of activity-based anorexia compared to *ad libitum* fed rats. This region is known for its predominant expression of corticotropin-releasing factor (CRF). Moreover, CRF mRNA expression was also reported to rise in the dorsomedial hypothalamic nucleus in rats with access to a running wheel (Kawaguchi et al., 2005). Interestingly, intracerebroventricular injection of the CRF antagonist, alpha-helical CRF attenuated the wheel-induced reduction of food intake and body weight (Kawaguchi et al., 2005) giving rise to a role of stimulated CRF signaling in activity-based anorexia. Lastly, this likely contributes to the increased circulating levels of corticosterone in rats (Burden et al., 1993) and cortisol in human anorexic subjects (Casper et al., 1979).

Lastly, also psychological parameters such as anxiety (Swinbourne and Touyz, 2007) and depressiveness (Debska et al., 2011) are often altered under conditions of AN. In the present study we observed an increased activation of the dorsal raphe nuclei and the raphe pallidus nucleus under conditions of activity-based anorexia, serotonergic nuclei that might play a role in the pathogenesis of depression (Michelsen et al., 2008). Interestingly, intraperitoneal injections of the serotonin agonist fenfluramine accelerated weight loss under conditions of activity-based anorexia compared to pair-fed controls giving rise to a mechanism other than reduced food intake (Atchley and Eckel, 2005). While the amygdala was not activated in the present study, the noradrenergic locus coeruleus showed a moderate activation in activity-based anorexia rats possibly leading to increased arousal (Aston-Jones and Waterhouse, 2016). Ascending projections might be involved in the observed activation of the paraventricular thalamic nucleus, the cingulate cortex and the hippocampus, brain structures involved in the processing of emotions and memory (Rolls, 2015). Interestingly, also the supraoptic nucleus as well as some magnocellular neurons of the paraventricular nucleus of the hypothalamus, two brain nuclei prominently expressing oxytocin, showed a robust activation in activity-based anorexia rats. It is important to note that oxytocin has been—besides its well-defined role during pregnancy—implicated in social memory, aggression and anxiety (Caldwell et al., 2016). Whether there is a direct link between anxiety or depressiveness and physical activity as suggested in humans (Holtkamp et al., 2004) warrants further investigation.

In summary, the activity-based anorexia model combines voluntary physical activity in a running wheel and time-restricted feeding to greatly reduce body weight. Interestingly, the food intake microstructure observed in activity-based anorexia rats did not differ from the one observed in the restricted feeding group arguing against a specific feeding phenotype. Also physical activity did not differ from the respective control group. Activity-based anorexia rats displayed an activation of distinct brain nuclei involved in the mediation of food intake, physical activity, thermoregulation as well as depression/anxiety and stress. Although these animal data have to be interpreted with caution, current data point toward the usefulness of the model to better understand pathophysiological alterations also occurring in AN.

## Author contributions

SS performed the experiments and drafted the manuscript. PP performed the experiments and analyzed the data. MGS and PK performed the experiments and reviewed the manuscript. TH wrote and reviewed the manuscript. MR gave critical input throughout the study and reviewed the manuscript. AS planned the experiments, analyzed the data and wrote the manuscript.

### Conflict of interest statement

The authors declare that the research was conducted in the absence of any commercial or financial relationships that could be construed as a potential conflict of interest.
